# Treatment of SLE: bridging the gap from clinical trials to the clinic - a meeting report

**DOI:** 10.1186/ar4219

**Published:** 2013-07-11

**Authors:** Leonard H Calabrese

**Affiliations:** 1 Cleveland Clinic Lerner College of Medicine of Case Western Reserve University, Department of Rheumatic and Immunologic Diseases, Orthopaedic and Rheumatologic Institute, Cleveland Clinic, Cleveland, OH, USA

## 

Systemic lupus erythematosus (SLE) is a complicated disease that frustrates many clinicians with its wide variety of presentations, signs, and symptoms. SLE is defined as a chronic disease characterized by inflammation of various organs, primarily the skin and joints. The disease does not have a clear etiologic picture. Immunologic abnormalities, highlighted by the production of various antinuclear antibodies, are a prominent feature. Patients with SLE suffer a variety of symptoms, most commonly related to the skin, musculoskeletal, hematologic, and serologic organs. In the recent past, a diagnosis of SLE implied a decreased lifespan resulting from internal organ system involvement or the toxic effects of therapy; however, recent improvements in research and clinical trials have provided clinical data that have dramatically enhanced survival in SLE patients. Nonetheless, SLE mortality rates remain a major concern.

Many clinicians in the field of rheumatology have substantial knowledge and practice gaps related to the diagnosis and care of patients with lupus. Even SLE experts disagree on points of SLE management. To address some of the issues, this supplement will present insights from internationally known experts in the field of lupus research and patient care.

The four abstracts in this supplement summarize live presentations at an educational symposium titled "Treatment of SLE: Bridging the Gap from Clinical Trials to Practice". Conceptually intertwined with the pathogenesis of SLE, the newly evolving topic of targeted therapy is the focus of this educational activity. A review of the current scientific implications for the biologic basis of SLE pathogenesis and the therapeutic management of the disease will increase rheumatologists' medical understanding of the molecular and cellular basis of SLE pathogenesis that is crucial to improving outcomes in these patient populations.

An adapted version of each of the full presentations given at the symposium is attached to each abstract. These adaptations bridge knowledge gaps on the latest SLE clinical research, biologic agents, and targeted therapies, as expert faculty compile and critically appraise new evidence and interpret its implications for improving the clinical management of patients with SLE.

The presentations are designed to address issues for clinical immunologists and rheumatologists who provide care for patients with SLE but who do not necessarily practice in the field of lupus. To that end, the expert faculty members provide analyses of the grueling and complex matrix of basic science and clinical science and then offer insight into how to translate those advances from the bench to the bedside.

The presentations start, appropriately, with a historical overview of SLE from its first reference during the 1100s, through the neoclassic period of the 1800s when it began to be defined, and into the modern era that began with the laboratory discovery of SLE cells. Weaved into this overview are descriptions of the most important contributions from the leading practitioners and researchers.

The faculty are among the most respected in the field. Bevra Hahn, MD, Professor Emerita of Medicine at the David Geffen School of Medicine at UCLA, has been in the field for a long time. She is a prolific author who has many contributions to medicine, including being the lead author on the American College of Rheumatology guidelines for screening, treatment, and management of lupus nephritis. Dr Hahn's presentation 'Unmet Needs: Therapeutic Standards of Care' focuses on the evidence-based best practices for SLE and how to fit the American College of Rheumatology guidelines into clinical care.

Michelle Petri, MD, MPH, Professor of Medicine at the Johns Hopkins University School of Medicine, Baltimore, MD, has organized several outstanding educational initiatives in lupus. She is lead author of the most recent classification criteria for lupus. In her presentation 'Measuring Disease Activity and Severity in Clinical Trials and the Clinic: Same or Different?' Dr Petri discusses the revised clinical and immunologic criteria for the classification of SLE, necessitated by substantial advances in knowledge of immunologic interplay of SLE. The question of when to monitor patients with stable disease has perplexed many clinicians, and Dr Petri describes evidence showing when specific variables usually can be detected and offers clinical guidance based on the evidence. She also presents a convincing argument for limiting the use of high-dose prednisone therapy, which has shown significant evidence of increased morbidity and mortality. To help those relying on prednisone, she describes some options found to be effective in head-to-head trials.

Daniel Wallace, MD, is a clinical leader and a highly respected clinical trial investigator. His presentation 'Clinical Impact of Biologic Therapy in the Treatment of SLE' is a thoroughly researched and referenced review of clinical trial data on biologic therapies in SLE, providing insights into the subtleties of the responder indexes, their impact on potential new therapies, and their potential use in clinical practice. He finishes with an in-depth review of belimumab, the clinical trial evidence base (including American College of Rheumatology and European League Against Rheumatology annual meetings and peer-reviewed literature), and his summary of its clinical implications for practice.

The goal of these presentations is to provide readers with a review the current evidence regarding SLE and translate it into implications for clinical practice. By providing the current best practices and addressing the identified knowledge and practice gaps, the supplement has the potential to substantially improve outcomes in this patient population.

## Competing interests

LHC has received consulting fees from Amgen, BMS, Centocor, Genentech/Roche, Pfizer, Sanofi Aventis and Savient.

